# Neurodevelopmental risks of SARS-CoV-2 infection in utero

**DOI:** 10.1038/s41390-025-04301-9

**Published:** 2025-07-18

**Authors:** Ariel Dunn, Atul Malhotra

**Affiliations:** 1 Department of Psychiatry, Monash University, Melbourne, VIC Australia; 2 Department of Paediatrics, Monash University, Melbourne, VIC Australia; 3 Monash Newborn, Monash Children’s Hospital, Melbourne, VIC Australia; 4 The Ritchie Centre, Hudson Institute of Medical Research, Melbourne, VIC Australia

Infection during pregnancy is a robust and well-established risk factor associated with adverse child developmental outcomes later in life, including developmental delays and neurodevelopmental disorders.^1^ In line with many other infections, a similar pattern is emerging as to the long-term detrimental effects of maternal SARS-CoV-2 infection on fetal neurodevelopment.^2^ Over 20 years of research suggests that the maternal immune response itself (termed maternal immune activation, MIA), rather than any specific pathogen, mediates disrupted inflammatory profiles and placental dysfunction via ‘cytokine storms’, leading to altered fetal brain development.^3^ The relative risk of inflammatory cytokine disruptions to neurodevelopmental outcomes is highly variable, with considerations for (but not limited to) gestational timing of exposure, severity of maternal inflammatory response, maternal age, and the child’s determined sex at birth.^4^ Early identification and intervention are crucial determinants of long-term neurodevelopmental outcomes across the lifespan, particularly for infants with developmental delays and those at a higher risk of neurodevelopmental disorders such as autism spectrum disorder (ASD) and attention-deficit hyperactivity disorder (ADHD). The ability to predict neurodevelopmental trajectories in infancy following maternal SARS-CoV-2 infection via specific inflammatory biomarkers is, therefore, a vital step in facilitating early identification and intervention in high-risk cases. However, such inflammatory biomarkers remain elusive, despite some recent studies investigating the role of inflammatory ‘cytokine storms’ on infant outcomes following maternal SARS-CoV-2 infection.^5^

In this issue of *Paediatric Research*, a study by Kehdi et al.^6^ investigated the long-term impact of maternal SARS-CoV-2 infection on maternal and infant peripheral biomarkers and behavioural outcomes, and whether these predicted developmental delays in infants up to 24 months old at a public maternal hospital in Fortaleza, Brazil, offering insights into the epidemiological landscape of a region with particularly high infection rates. This elegantly designed study included 15 mother-infant dyads at 35-37 weeks gestation who had tested positive for SARS-CoV-2 infection when admitted for delivery. A comprehensive cytokine and chemokine assessment was conducted on maternal and cord serum samples collected within 24 h of delivery, which were matched by gestational age to a small number of control samples given the high rates of infection during the study (January 2021–March 2022). Infants were followed up at 6- and 24-months-old, with infant blood samples and clinical neurodevelopmental assessments undertaken by trained and blinded research assistants, utilising the Bayley’s Scale of Infant and Toddler Development 3^rd^ Ed. (BSID-III) at each time point, with a focus on key developmental domains of cognition, communication (expressive and receptive) and motor skills.

The findings of this study were informative, albeit sobering, particularly given the overarching lack of inclusive and representative infant health data from this region. A complex immune profile was characterised in SARS-CoV-2 infected pregnant women, with robust and sustained increases in pro-inflammatory and chemotactic cytokines (including IL-19, IL-23, IL-8, CXCL10), and decreases in anti-inflammatory cytokines (such as VEGF, MIF, IGFBP-3), indicative of a hyperinflammatory gestational environment, coupled with altered immune regulatory pathways. Many of these cytokines cross the placental barrier.^7^ Indeed, increased maternal IL-8 at birth was specifically associated with motor and communication delays in 24-month-old infants, substantiating the adverse implications of MIA for fetal neurodevelopment. This finding aligns with a recent prospective longitudinal study investigating child developmental trajectories following severe maternal SARS-CoV-2 infection during pregnancy, with elevated maternal IL-6 and IL-17A associated with poorer problem-solving and communication skills in 12-month-old infants.^5^ Likewise, animal models of MIA consistently demonstrate that pro-inflammatory cytokines, particularly IL-6 are key mediators of disrupted neurodevelopmental processes.^8^ Infants born to SARS-CoV-2 infected mothers also showed disrupted and sustained inflammatory markers at birth (cord blood) and 6-months, including IFN-γ, TNFα, IL-6, CCL3 and CXCL10, which were associated with developmental delays in approximately half of the studied infants at 24-months-old.

Two major strengths of the Kehdi et al. study^6^ are clear, despite the modest sample size. One is the prospective longitudinal design, with multiple infant timepoints assessed at birth, 6- and 24-months in the same study participants. Developmental delays particularly in the communication domain were more frequently observed at 24-months versus 6-months in infants exposed in utero to SARS-CoV-2 infection, a finding strengthened by the BSID-III tools’ extended use in the early identification of language disabilities in young children.^9^ The number of longitudinal studies linking maternal SARS-CoV-2 infection during pregnancy to child developmental outcomes are increasing, however, many lack biological samples, which is another major strength of this study. The collection of blood serum samples from three sources—maternal, umbilical cord, and infants at both timepoints—directly enables the identification of distinct cytokine and chemokine immune biomarkers, and their association with specific developmental milestones in young infants. The altered cytokine profiles in the umbilical cord and the blood of infants exposed to late-gestation SARS-CoV-2 infection *in utero* at 6 months of age could therefore be used as predictors of their developmental trajectories later in life.

It is important to note that child developmental trajectories are extremely heterogenous, in part due to the variability of factors discussed previously such as timing of exposure and infection severity. Therefore, not all children exposed to altered inflammatory processes in utero, including from SARS-CoV-2 infection, will have developmental delays and/or neurodevelopmental disorders such as ASD or ADHD. Therefore, animal or cell models of prenatal SARS-CoV-2 infection^10^ provide highly valuable tools to control for such aetiological variability, to elucidate the underlying mechanisms of MIA-induced neurodevelopmental disruptions. These models also provide an opportunity to disentangle the relationship between peripheral biomarkers such as pro-inflammatory and chemotactic cytokines, and brain alterations relevant to neurodevelopmental processes. Future longitudinal studies of neurodevelopmental outcomes following in utero SARS-CoV-2 exposure would also greatly benefit from large sample sizes allowing sufficient statistical power to identify sex differences in altered neurodevelopmental trajectories. Whilst sex-as-a biological-factor is an important variable to consider in all fields of research, it is particularly relevant to child neurodevelopmental outcomes given the growing clinical and scientific understanding of distinct differences in symptom expression, lived experience, and likely neurobiology between males and females with neurodevelopmental disorders such as ASD and ADHD.^11^

The findings from this study highlight the importance of long-term monitoring of child developmental trajectories following exposure to SARS-CoV-2 infection during pregnancy, as well as the complex interplay between the maternal-placental-fetal environment during gestation. The robust and established risk of disrupted inflammatory states within the gestational environment emphasises the critical need for preventative strategies during pregnancy to mitigate both short- and long-term ramifications on developmental outcomes in children.
